# Elevated hypertriglyceridemia and decreased gallstones in the etiological composition ratio of acute pancreatitis as affected by seasons and festivals: A two-center real-world study from China

**DOI:** 10.3389/fcimb.2022.976816

**Published:** 2022-11-24

**Authors:** Wenhua He, Gang Wang, Bingjun Yu, Liang Xia, Yong Zhu, Pi Liu, Hua Chen, Rui Kong, Yin Zhu, Bei Sun, Nonghua Lu

**Affiliations:** ^1^ Department of Gastroenterology, Pancreatic Disease Centre, The First Affiliated Hospital of Nanchang University, Nanchang, China; ^2^ Department of Pancreatic and Biliary Surgery, The First Affiliated Hospital of Harbin Medical University, Harbin, China; ^3^ Key Laboratory of Hepatosplenic Surgery, Ministry of Education, The First Affiliated Hospital of Harbin Medical University, Harbin, China

**Keywords:** acute pancreatitis, epidemiology, season, etiology, hypertriglyceridemia

## Abstract

**Objective:**

The purpose of this study was to observe the annual variation in the etiology of acute pancreatitis (AP) and its relationship with seasons and festivals.

**Methods:**

From 2011 to 2017, 5146 adult patients with AP were studied, including 4110 patients from the First Affiliated Hospital of Nanchang University (South center) and 1036 patients from the First Affiliated Hospital of Harbin Medical University (North center). We analyzed the overall annual variation in the etiology of AP and then compared the differences in etiology between the two regions, as well as the effects of seasons and festivals on the etiology of AP.

**Results:**

Gallstones, hypertriglyceridemia (HTG) and alcohol were the top three etiologies of AP. Gallstone AP showed a downward trend (P<0.001), and HTG-AP and alcohol AP showed an upward trend (both P<0.01). Among the etiologies of AP, gallstones and HTG were affected by seasons and festivals. The composition ratio of HTG-AP increased, while gallstone AP decreased in winter and in months with long holidays (all P<0.01). The composition ratio of gallstone AP in the south center was higher than that in the north center (59.5% vs. 49%), especially in summer (62.9% vs. 44.0%) and autumn (61.5% vs. 45.7%, all P<0.001).

**Conclusions:**

The composition ratio of HTG-AP increased while gallstone AP decreased in the past 7 years, and they were affected by seasons and festivals.

## Introduction

Acute pancreatitis (AP) is a common gastrointestinal disease that is associated with substantial suffering and morbidity (Forsmark et al., 2016). The incidence of AP has been increasing in recent years worldwide (Lindkvist et al., 2004; Spanier et al., 2013; Peery et al., 2015; Peery et al., 2019). In the past 10 years, the number of hospitalized patients with AP in the United States has increased by at least 20% (Peery et al., 2015; Peery et al., 2019). Migrating gallstones cause transient obstruction of the pancreatic duct, and exposure of the pancreas to biliary constituents represents the most common cause of AP (Forsmark et al., 2016). Alcohol is the second most common cause of AP and accounts for approximately 30% of cases in Western countries (Gullo et al., 2002; Yadav and Lowenfels, 2013; Forsmark et al., 2016); however, it only accounts for approximately 5% of all cases in China (Zhu et al., 2017; Wu et al., 2017). Hypertriglyceridemia (HTG) is a well-established cause of AP (Rawla et al., 2018), and it has become the second leading cause of AP in China (Zhu et al., 2017; Mukherjee et al., 2019). It is worth evaluating the common regional characteristics and variation in the main causes of AP in China.

We speculate that the seasonal climates and food cultures of different countries are the main reasons for the differences in etiology. Seasonality is a well-known factor in the epidemiology of many diseases, such as peptic ulcers (Bendahan et al., 1992) and inflammatory bowel disease (Koido et al., 2013). A study from Germany explored the connection between AP and seasonality and found that there was no seasonal pattern in AP occurrence (Lankisch et al., 1998). However, subsequent studies have shown that there are periodic changes in the onset of AP, that gallstone and alcohol AP significantly increase in spring in Italy (Gallerani et al., 2004) and that alcohol AP is highest in summer and autumn in Finland (Räty et al., 2003). The occurrence of alcoholic AP also increases significantly during holidays. (Räty et al., 2003; Roberts et al., 2013)^ ^A recent study from eastern China found that the prevalence of AP is significantly higher in spring and autumn, especially for gallstone and HTG-induced AP (HTG-AP) (Wu et al., 2017). However, China is a vast territory, and different regions (such as the north and south) have different climates and diets. Both the composition ratio and trend of the etiologies of AP in these places are still unclear. This real-world, dual-center study collected data from AP patients from two pancreatic disease centers to observe changes in the main causes of AP in China over the past 7 years, along with the relationship of AP with seasons and festivals.

## Methods

### Patients and locations

This dual-center, real-world retrospective cohort study was conducted at two pancreatic disease centers. The study was approved by the Medical Ethics Research Committee of the First Affiliated Hospital of Nanchang University (South center) and the Medical Ethics Research Committee of the First Affiliated Hospital of Harbin Medical University (North center). We selected AP patients with onset within 7 days from January 2011 to December 2017 from the AP databases of the two pancreatic disease centers. We excluded the following patients: 1) patients aged < 18 years, 2) patients transferred from another tertiary hospital, 3) patients with recurrent pancreatitis, 4) patients with an acute attack of chronic pancreatitis, and 5) patients with pancreatic cancer diagnosed after AP onset. The diagnosis and classification were based on the revised Atlanta classification. The diagnostic criteria for the various etiologies of AP are shown in **S-Table 1**.

### Classification of seasons and festivals

In China, the four seasons are determined based on the combination of astronomical seasons and climate seasons: spring (March-May), summer (June–August), autumn (September–November), and winter (December–February). There are three statutory long holidays to celebrate festivals in China: the Spring Festival in February, the labor day in May and the National Day in October. People hold various banquets (such as wedding banquets, etc.), family reunions and dinners among friends in these three months.

### Data collection and statistical analysis

Data on demographics, AP etiology, laboratory indicators, scoring systems, local and systemic complications, and clinical outcomes of patients admitted to the hospital from January 2011 to December 2017 were collected and analyzed. Demographic and baseline characteristics were analyzed using descriptive analysis. Qualitative variables were described using numbers and percentages. Quantitative variables are presented as the mean ± standard deviation (SD). The median and interquartile range (IQR) were reported if the distribution of the variable was not normal. A t test was performed for continuous variables when the data were normally distributed, and the Kruskal–Wallis test was performed when the data were not normally distributed. The *X (* Lindkvist et al., 2004 *)* test was performed for categorical variables, and relative risk was calculated for dichotomous variables. A two-tailed *P* < 0.05 was considered to indicate statistical significance.

## Results

### Participant characteristics

During the 7-year study period, 5146 patients with AP were examined, with a median age of 49 years, and 57.2% were male. According to the revised Atlanta severity classification, the numbers of mild, moderate, and severe cases were 1914 (37.1%), 2382 (46.1%), and 849 (16.4%), respectively. The median length of hospital stay was 8 days, and the mortality rate was 1.4% (**Table 1**). Of the 5,146 patients, 4,110 were hospitalized in the South center, and 1,036 were hospitalized in the North center. Compared with patients in the South center, patients with AP in the North center were younger [median 46 years (IQR 37-58) vs. 50 years (IQR 40-63), P = 0.00] and tended to be male (63.68% vs. 55.8%, P = 0.00). More AP patients from the North center had a history of smoking (34.3% vs. 18.6%, P = 0.00) and drinking (38.7% vs. 17.6%, P = 0.00) than patients from the South center. In terms of coexisting conditions, more patients had coronary heart disease (7.0% vs. 1.8%, P = 0.00), diabetes (13.3% vs. 8.8%, P = 0.00) and cerebral infarction (4.4% vs. 1.1%, P = 0.00) in the North center than in the South center.

**Table 1:** Baseline Characteristics of the Patients.

Characteristic	Total	South center	North center	P value
	5146	n=4110	n=1036
Age, median (IQR), y	49 (39-63)	50 (40-63)	46 (37-58)	<0.001
Male sex, no. (%)	2952 (57.2)	2293 (55.8)	659 (63.68)	<0.001
Smoking history, no. (%)	1119 (21.7)	764 (18.6)	355 (34.3)	<0.001
Drinking history, no. (%)	1124 (21.8)	723 (17.6)	401 (38.7)	<0.001
Coexisting condition, no. (%)				
Coronary heart disease, no. (%)	146 (2.8)	73 (1.8)	73 (7.0)	<0.001
Hypertension	891 (17.3)	700 (17.0)	191 (18.4)	0.29
COPD, no. (%)	49 (1.0)	47 (1.1)	2 (0.2)	0.17
Diabetes, no. (%)	498 (9.6)	360 (8.8)	138 (13.3)	0
Cirrhosis, no. (%)	21 (0.4)	17 (0.4)	4 (0.4)	0.87
Chronic renal failure, no. (%)	9 (0.2)	9 (0.2)	0 (0)	0.28
Cerebral infarction, no. (%)	92 (1.8)	46 (1.1)	46 (4.4)	<0.001
WBC (IQR),	12.0 (8.7-15.7)	12.0 (8.5-15.4)	12.3 (9.0,16.3)	<0.001
HCT (IQR),	40.0 (36.0,44.0)	40.0 (35.9,44.0)	41.0 (37.4,46.1)	<0.001
AMY (IQR),	251 (87,703)	279 (90,742)	184 (82,531)	<0.001
APACHEII (IQR),	6 (3,8)	6 (3,9)	5 (3,7)	<0.001
Disease severity				
MAP, no. (%)	1914 (37.1)	1485 (36.1)	429 (41.4)	0.01
MSAP, no. (%)	2382 (46.1)	1892 (46.0)	490 (47.3)	0.68
SAP, no. (%)	849 (16.4)	732 (17.8)	117 (11.3)	<0.001
Rate of ICU admission	1160 (22.5)	1045 (25.4)	115 (11.1)	<0.001
Duration of hospitalization (IQR),	8 (5,12)	8 (5,12)	9 (6,13)	0.001
Death, no. (%)	72 (1.4)	58 (1.4)	14 (1.4)	0.5

AMY, amylase; APACHE II, Acute Physiology and Chronic Health Evaluation II; COPD, Chronic obstructive pulmonary disease; HCT, Hematocrit; IQR, interquartile range; MAP, mild acute pancreatitis; MSAP, moderately severe acute pancreatitis; SAP, severe acute pancreatitis; WBC, White Blood Cell. The data collection time points for WBC, HCT, AMY, and APACHEII were within 24 hours of admission.

### Etiology of AP

**Table 2** shows that gallstones, HTG and alcohol were the top three etiologies of AP in both the north and south centers, and the total composition ratios of these causes were 57.4%, 23.9%, and 8.8%, respectively. Gallstone AP was higher, and idiopathic pancreatitis was lower in the south center than in the north center (59.5% vs. 49%, 6.1% vs. 14.2%, respectively, both P <0.001). In the south center, gallstone AP was more common in female and elderly patients, and HTG and alcohol AP were more common in male and young patients (all P<0.001, **S-Table 2**). In the North center, there was no sex difference in HTG-AP (P = 0.70, **S-Table 2**) and no age difference in alcohol AP (P = 0.09, **S-Table 2**).

**Table 2:** Etiological composition of acute pancreatitis in South and North center.

Characteristic	Total	South center	North center	P value
	n=5146	n=4110	n=1036
Biliary, no. (%)	2952 (57.4)	2444 (59.5)	508 (49.0)	<0.001
Alcohol abuse, no. (%)	452 (8.8)	349 (8.5)	103 (9.9)	0.14
HTG, no. (%)	1231 (23.9)	989 (24.1)	242 (23.4)	0.65
Operative^#^, no. (%)	17 (0.3)	8 (0.2)	9 (0.9)	0.03
PEP, no. (%)	22 (0.4)	20 (0.5)	2 (0.2)	0.15
Traumatic, no. (%)	12 (0.2)	10 (0.2)	2 (0.2)	0.55
Autoimmunity, no. (%)	7 (0.1)	1 (0.02)	6 (0.6)	<0.001
Parapapillary diverticulum of duodenum, no. (%)	17 (0.4)	17 (0.4)	0 (0)	0.02
Hypercalcaemia , no. (%)	16 (0.3)	2 (0.05)	14 (1.4)	<0.001
Drug-induced , no. (%)	15 (0.3)	15 (0.4)	0 (0)	0.03
Other etiologies*, no. (%)	7 (0.1)	4 (0.1)	3 (0.3)	0.13
Idiopathic , no. (%)	398 (7.7)	251 (6.1)	147 (14.2)	<0.001

HTG, Hypertriglyceridaemia; PEP, post-ERCP Pancreatitis. ^#^It refers to the acute pancreatitis after surgery. *Other etiologies: pancreatic cancer, ampullary cancer, papillary sphincter dysfunction, pancreatic Division.

### Annual variation in major etiologies of AP

**Figure 1:**
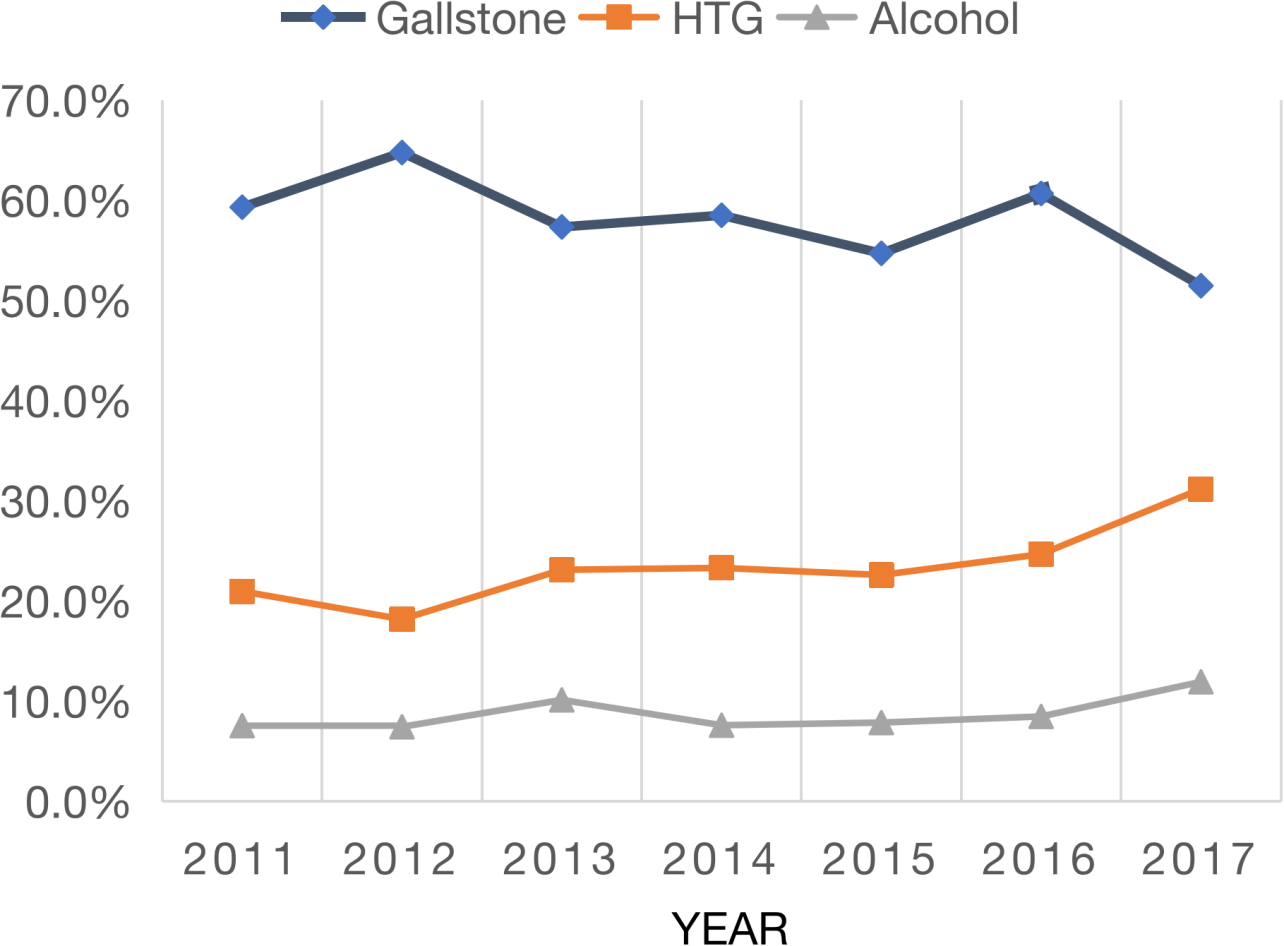
Annual variation in major etiologies of acute pancreatitis.

**Figure 1** shows the trends of the top three etiologies of AP from 2011 to 2017. Gallstone AP showed a downward trend (P<0.001), but HTG-AP and alcohol AP showed an upward trend (P<0.001 and P = 0.006, respectively). We further compared the changes in etiology before and after 2015 and found that the composition ratio of gallstone AP in the South center decreased (60.2% vs. 55.1%, P<0.001, **S-Table 3**); HTG-AP increased (21.3% vs. 26.0%, P<0.001), but alcohol AP did not change significantly. The changes in the abovementioned etiological composition in the North center were consistent with those in the South center.

### Seasonal variation in major etiologies of AP

**Figure 2:**
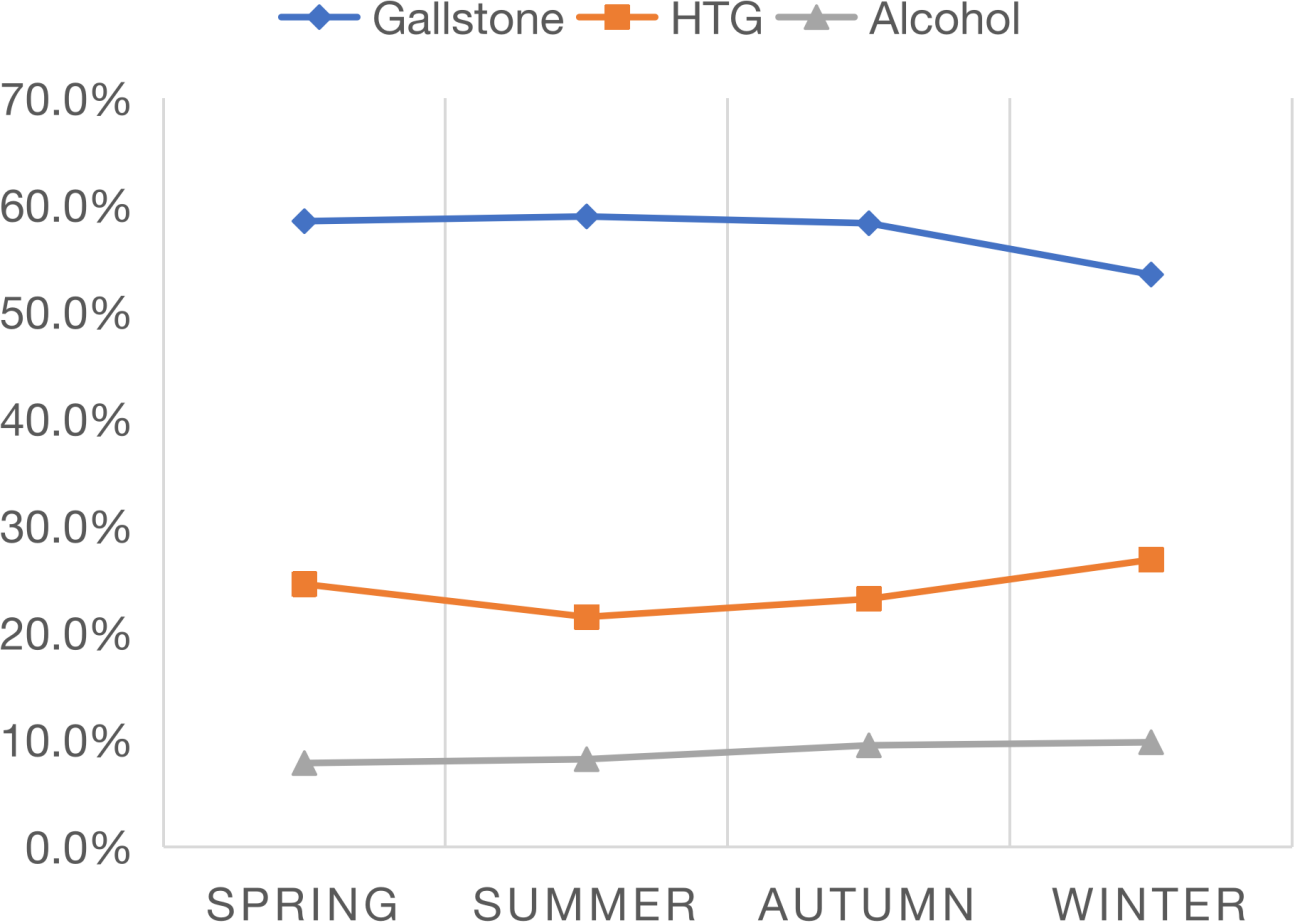
Seasonal variation in major etiologies of acute pancreatitis.

**Figure 2** shows that the composition ratio of the three major causes changes seasonally. In winter, the composition ratio of HTG-AP increased, while gallstone AP decreased (both P < 0.05), but alcohol AP did not show seasonal changes (**Figure 2**). There were seasonal differences in the etiological composition ratio between the two centers: the gallstone AP in the North center in summer and autumn was significantly lower than that in the South center (44.0% vs. 62.9%, 45.7% vs. 61.5%, respectively, both P <0.001, **Table 3**). In summer, the HTG-AP was lower in the North center than in the South center (17.2% vs. 22.6%, P = 0.04).

**Table 3:** The composition of AP etiology in different seasons in in South and North center.

Etiology	season	South center		North center		P-value
		(n)	constituent ratio	(n)	constituent ratio	
Gallstone	Spring	603	59.4%	131	54.6%	0.37
	Summer	687	62.9%	128	44.0%	0.00
	Autumn	627	61.5%	118	45.7%	0.00
	Winter	527	53.6%	131	53.0%	0.87
HTG	spring	247	24.3%	61	25.4%	0.67
	summer	247	22.6%	50	17.2%	0.04
	autumn	234	22.9%	62	24.0%	0.71
	winter	261	26.6%	69	27.9%	0.66
Alcohol	spring	82	8.1%	16	6.7%	0.48
	summer	84	7.7%	29	10.0%	0.21
	autumn	90	8.8%	31	12.0%	0.12
	winter	93	9.5%	27	10.9%	0.49

HTG, Hypertriglyceridaemia.

### Monthly variation in major etiologies of AP and the impact of festivals

**Figure 3:**
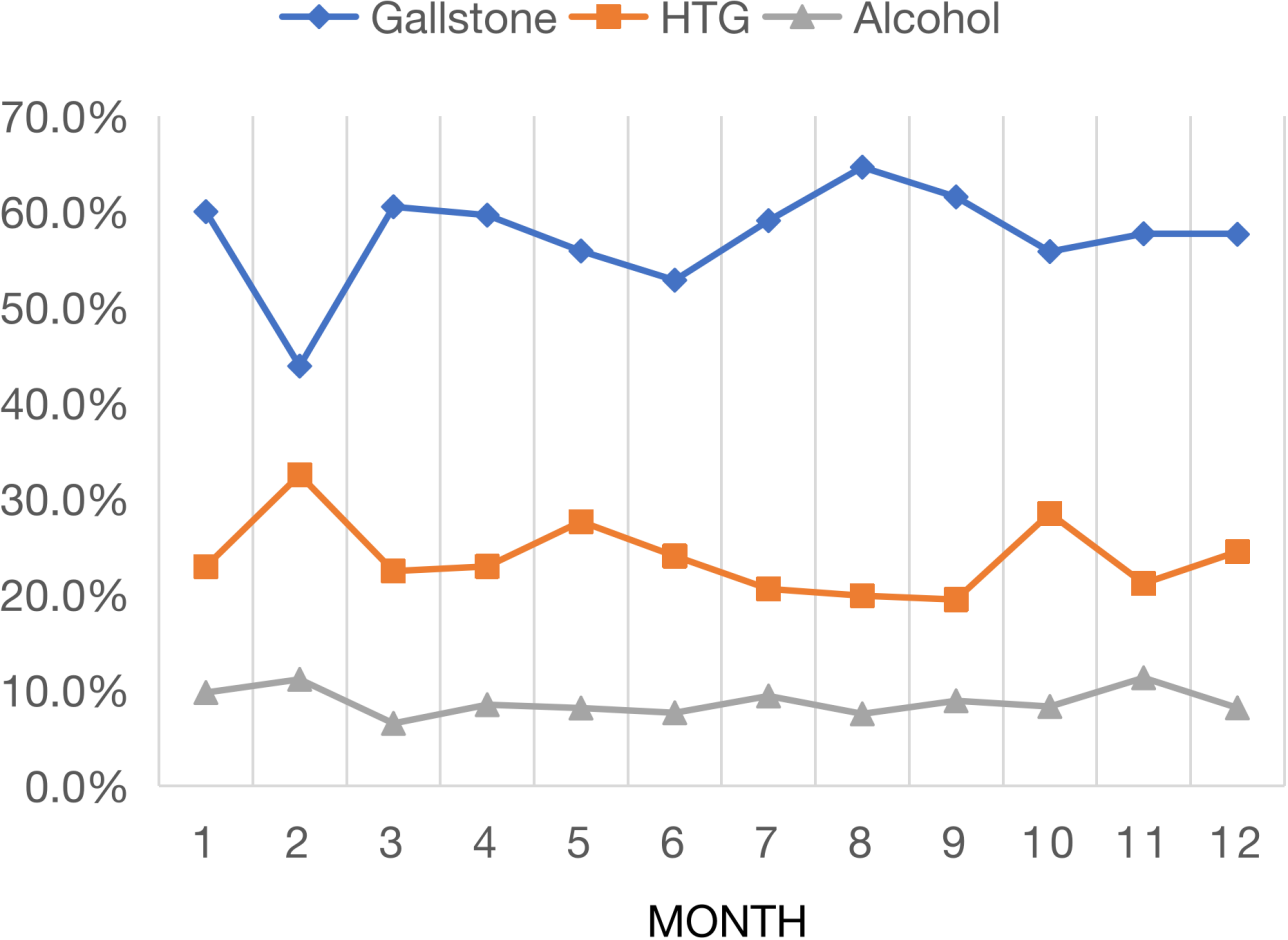
Monthly variation in major etiologies of acute pancreatitis.

**Figure 3** shows the monthly variation trend of the composition ratio of the three major causes of AP, in which HTG-AP increased significantly, but gallstone AP decreased significantly in February, May and December (both P <0.001). Alcohol AP showed no significant monthly change. The composition ratio of gallstone AP was lower in months with long holidays than in months without long holidays (52.0% vs. 59.3%, P<0.001, **S-Table 4**). In contrast, HTG-AP was higher in months with long holidays than in months without long holidays (29.5% vs. 21.9%, P<0.001), but alcohol AP was not significantly elevated in long holiday months (P=0.564).

## Discussion

This study explored the variation in the etiology of AP at two pancreatic disease centers in China and found that gallstones, HTG and alcohol were the top three etiologies of AP. HTG-AP and alcohol AP showed an upward trend, while gallstone AP showed a downward trend. In addition, the composition ratios of gallstone AP and HTG-AP were affected by seasons and festivals. Compared with patients with AP in the South center, patients with AP in the North center were younger and tended to be male, and more patients had a history of smoking and drinking. In terms of comorbidities, AP patients with coronary heart disease, diabetes and cerebral infarction were more likely to be found in the North center than in the South center. The geographical environment, food culture, and dietary habits are substantially different between the southern and northern regions in China, and alcohol and Western eating patterns are more prevalent in the northern region (Song and Cho, 2017).

Although gallstones were the dominant etiology in the two regions, the composition of gallstones AP was higher in the south center than in the north center. Regional differences in gallstone AP have also been reported in Europe, with the highest proportion of gallstone AP in southern Europe and the lowest in eastern Europe (Roberts et al., 2017). We believe that the higher proportion of male and younger patients in the North center resulted in a lower proportion of gallstone APs in the North center than in the South center. Both sex and age have an important influence on the occurrence of gallstone AP; the risk of gallstones is twice as high in women than in men, and age further increases the risk for gallstone AP in both sexes (Shaffer, 2005). Previous studies have also shown that gallstone AP is more common in females and that its occurrence increases sharply with age (Roberts et al., 2013; Zhu et al., 2017; Wu et al., 2017). The composition ratio of alcohol AP in the South and North centers was 8.5% and 9.9%, respectively, which was lower than that in Europe and the United States (Yadav and Lowenfels, 2013; Roberts et al., 2017). Additionally, alcohol AP was more common in male and young patients, which is consistent with the results of studies from the United Kingdom and the United States (Yadav and Lowenfels, 2013; Roberts et al., 2013).

This study shows that HTG accounts for 23.9% of the etiological composition of AP, which is different from Western reports (Forsmark et al., 2016). HTG is a rare cause in European and American countries, accounting for approximately 2–5% of AP cases (Forsmark et al., 2016). However, two systematic reviews reported that HTG is the third most common cause of AP, accounting for 10% of cases worldwide (Carr et al., 2016; Adiamah et al., 2018). In China, with the development of the economy, 33.97% of adult residents developed dyslipidemia in 2010, almost twice the number in 2002 (Pan et al., 2016). Correspondingly, studies from China have shown that HTG was the second leading cause of AP (10.4%-14.3%) in 2010 (Zheng et al., 2015; Zhu et al., 2017). The increase in the proportion of HTG AP may lead to a relative decrease in gallstone AP. Gallstone AP decreased by 5%, consistent with a 5% increase in HTG in the South center after 2015. HTG is the main type of dyslipidemia in China, but hypercholesterolemia is the main type in Western countries (Pan et al., 2016). This may be the reason why the incidence rate of HTG-AP in China is higher than that in Western countries.

Seasonal variation in the etiology of AP remains uncertain. This study showed that the composition ratio of gallstone AP was significantly lower than in other seasons, which suggests that rising temperatures may increase the incidence of gallstone AP. A cross-sectional study found that the frequency of gallstone-induced acute cholecystitis is higher in summer than in other seasons (Hosseini et al., 2006). Cholecystectomy is also related to periodic changes in temperature, with the highest number of cases occurring in summer and the lowest in winter (Liu et al., 2014). In addition, the lower composition ratio of gallstones AP in the north center in summer and autumn may be related to the lower temperature in northern China than in southern China in summer and autumn. The composition ratio of HTG-AP was the lowest in summer and the highest in winter, which is related to Chinese residents eating more high-fat food in winter. A high-fat diet is often considered a common secondary cause of hypertriglyceridemia and a risk factor for AP (Scherer et al., 2014). There was no significant seasonal change in alcoholic AP, which is consistent with data from eastern China (Wu et al., 2017).

Two studies from Europe found that increased alcohol consumption in months with holidays leads to the highest prevalence of acute alcoholic AP but not gallstone AP (Räty et al., 2003; Roberts et al., 2013). Our research shows that the composition ratio of HTG-AP was significantly higher and gallstone AP was relatively lower in months with long holidays. The long holidays are traditional Chinese family reunion festivals where relatives and friends frequently gather for meals and are more likely to overeat, consume a high-fat diet and abuse alcohol, leading to increased serum triglyceride levels and an increase in the prevalence of HTG-AP, which is consistent with a study from Shanghai, China (Wu et al., 2017). We speculate that the increase in the proportion of HTG led to a relative decrease in gallstone AP. Moreover, long holidays mainly occur in spring and autumn, and the incidence of gallstone disease is lower in cold climates. Given the increasing incidence of HTG-AP, it has become a major issue in China. Health education should emphasize a low-fat diet and alcohol abstinence, especially during festivals. The formulation of health policies should take into account the influence of regions, seasons and festivals.

The advantage of this study is that a large sample of cases was collected, including more than 5000 patients with AP from two pancreatic centers in southern and northern China. A limitation, however, is that these data do not fully represent all AP patients in these two regions. This selection bias may have been relatively small because most AP patients in lower-level hospitals are transferred to tertiary hospitals for better treatment.

In conclusion, our study revealed that the composition ratio of HTG-AP increased both in the south and north centers, while gallstone AP showed a downward trend. The etiology of AP was also affected by seasons and festivals. In winter and months with long holidays, the composition ratio of HTG-AP increased, and gallstone AP decreased significantly. Climate, a high-fat diet and alcohol consumption may be the main causes of the variation in the etiology of AP during seasons and holidays.

## Data availability statement

The raw data supporting the conclusions of this article will be made available by the authors, without undue reservation.

## Author contributions

WH: study design, statistical analysis, and manuscript preparation; GW: data collection, reviewed and revised the manuscript; BY: data abstraction and collection and manuscript preparation; LX: data collection; YoZ: data collection; PL: data collection; YiZ: data collection and data analysis; BS: study design, reviewed and revised the manuscript; NL: study design, reviewed and revised the manuscript. All authors contributed to the article and approved the submitted version.

## Funding

This research was funded by the National Key Clinical Specialist Project [Award number: (2011)872], National Natural Science Foundation of China (81860122), Jiangxi Province Outstanding Youth Talent Funding Program (20192BCBL23021), National Nature Scientific Foundation of China (82070657), Applied Technology Research and Development Project of Heilongjiang Province (GA20C019), Outstanding Youth Funds of the First Affiliated Hospital of Harbin Medical University (HYD2020JQ0006), and Research Projects of Chinese Research Hospital Association (Y2019FH-DTCC-SB1).

## Conflict of interest

The authors declare that the research was conducted in the absence of any commercial or financial relationships that could be construed as a potential conflict of interest.

The reviewer GL declared a past co-authorship with the author YiZ to the handling editor.

## Publisher’s note

All claims expressed in this article are solely those of the authors and do not necessarily represent those of their affiliated organizations, or those of the publisher, the editors and the reviewers. Any product that may be evaluated in this article, or claim that may be made by its manufacturer, is not guaranteed or endorsed by the publisher.
