# Supplementary material for: Elevated hypertriglyceridemia and decreased gallstones in the etiological composition ratio of acute pancreatitis as affected by seasons and festivals: A two-center real-world study from China
Source: Front Cell Infect Microbiol. 2022 Nov 24;12:976816. doi: 10.3389/fcimb.2022.976816 (PMC9730824; doi:10.3389/fcimb.2022.976816)
Supplement: Supplementary file 1 [file Table_1.docx]

**S-Table1：Definition of main etiologies of acute pancreatitis**

| Define terms | Definition | Reference |
| --- | --- | --- |
| Gallstone | A biliary etiology was defined as the presence of dilated CBD, or two of the following three laboratory abnormalities: (1) serum bilirubin concentration > 1.9 mg/d; (2) ALT activity > 100 U/L with an ALT activity higher than the AST activity; and (3) AKP > 195 U/L with a GGT activity > 45 U/L | Endoscopy 2011; 43: 8-13 |
| Alcohol | Patient has a history of over 5 years of heavy alcohol consumption (> 50 g per day) | Am J Gastroenterol. 2013;108(9):1400-1415 |
| HTG | Serum TG rise above 1,000 mg / dl, or serum TG > 500 mg / dl in the absence of gallstones and / or history of significant history of alcohol use/ or other known cause of AP. | Am J Gastroenterol.2013;108(9):1400-1415. |
| Idiopathic | AP Patient with no etiology established after initial laboratory (including lipid and calcium level) and imaging tests (transabdominal ultrasound, CT, EUS and MRCP in the appropriate patient) | Am J Gastroenterol. 2013;108(9):1400-1415. |

AP, acute pancreatitis; HTG, hypertriglyceridaemia; CBD, common bile duct; CT, computed tomography; EUS, endoscopic ultrasonography; ALT, alanine aminotransferase; AST, aspartate aminotransferase; AKP, alkaline phosphatase; GGT, γ-glutamyltransferase; TG, triglycerides; MRCP, magnetic resonance cholangiopancreatography.

**S-Table 2：The influence of patient's gender and age on the etiology of acute pancreatitis**

|  | South center | | | | North center | | | |
| --- | --- | --- | --- | --- | --- | --- | --- | --- |
|  | Case, n | gallstone | HTG | Alcohol | Case, n | gallstone | HTG | Alcohol |
| Sex |  |  |  |  |  |  |  |  |
| Male | 2293 | 48.70% | 30.90% | 14.60% | 659 | 44.90% | 23.80% | 14.30% |
| Female | 1817 | 73.00% | 15.40% | 0.80% | 377 | 56.20% | 22.50% | 2.40% |
| P value |  | <0.001 | <0.001 | <0.001 |  | <0.001 | 0.70 | <0.001 |
| Age,y |  |  |  |  |  |  |  |  |
| ≤50 | 2069 | 39.00% | 39.90% | 11.60% | 629 | 42.40% | 29.10% | 11.30% |
| >50 | 2041 | 80.20% | 8.00% | 5.30% | 407 | 59.20% | 14.50% | 7.90% |
| P value |  | <0.001 | <0.001 | <0.001 |  | <0.001 | <0.001 | 0.09 |

**S-Table 3．Etiologies of AP in the South and North center before and after 2015.**

| Etiology | South center | | | North center | | |
| --- | --- | --- | --- | --- | --- | --- |
|  | 2011-2014  n=1933 | 2015-2017  n=2177 | P value | 2011-2014  n=351 | 2015-2017  n=685 | P value |
| Gallstone, no. (%) | 1375 (60.2%) | 1577 (55.1%) | <0.001 | 1206 (62.4%) | 1238(56.9%) | <0.001 |
| Alcohol, no. (%) | 184 (8.1%) | 268 (9.4%) | 0.055 | 149 (7.7%) | 200 (9.2%) | 0.09 |
| HTG, no. (%) | 487 (21.3%) | 744 (26.0%) | <0.001 | 391 (20.2%) | 598 (27.5%) | <0.001 |

**S-Table 4. The etiological composition ratio of AP in months with and without long holidays**

|  | Long holidays | No Long holidays | P |
| --- | --- | --- | --- |
| Total |  |  |  |
| Gallstone | 703(52.00%) | 2249(59.30%) | 0.000 |
| HTG | 399(29.50%) | 832(21.90%) | 0.000 |
| Alcohol | 124(9.20%) | 328(8.60%) | 0.564 |
| South center |  |  |  |
| Gallstone | 582(53.30%) | 1862(61.70%) | 0.000 |
| HTG | 314(28.80%) | 675(22.40%) | 0.000 |
| Alcohol | 101(9.30%) | 248(8.20%) | 0.290 |
| North Center |  |  |  |
| Gallstone | 121(46.20%) | 387(50.00%) | 0.000 |
| HTG | 85(32.40%) | 157(20.30%) | 0.000 |
| Alcohol | 23(8.80%) | 80(10.30%) | 0.467 |
